# Editorial: Hair cells: From molecules to function, volume II

**DOI:** 10.3389/fncel.2022.1046646

**Published:** 2022-10-13

**Authors:** Mary Ann Cheatham, Bernd Fritzsch, David Z. He, Bradley J. Walters

**Affiliations:** ^1^The Knowles Hearing Center, Communication Sciences and Disorders, Northwestern University, Evanston, IL, United States; ^2^Department of Biology, The University of Iowa, Iowa City, IA, United States; ^3^Department of Otolaryngology, The University of Iowa, Iowa City, IA, United States; ^4^Department of Biomedical Sciences, Creighton University, Omaha, NE, United States; ^5^Department of Otolaryngology - Head and Neck Surgery, University of Mississippi Medical Center, Jackson, MS, United States

**Keywords:** cochlea, vestibular system, hearing, balance, auditory, supporting cell, hair cell

Now that the human genome has been sequenced (International Human Genome Sequencing Consortium, 2004; Nurk et al., 2022), genetic medicine is growing rapidly (Miller, 1992) and there is no reason that hearing health should not be a part of this movement. Although the opportunities for dealing with hearing loss and deafness have never been more compelling, we must define underlying disease mechanisms and identify biomarkers for patient susceptibility. This effort requires a better characterization of the normal system and how it develops in order to provide a foundation for learning how the system can be repaired and how hearing loss can be ameliorated.

This second volume continues to expand our knowledge of hair cell structure and specifically the hair bundle at the apical end of the sensory receptor cells in the inner ear. O'Donnel and Zheng report that *Camsap3*, a microtubule minus-end regulator, is required to form the kinocilia. While kinocilia do not persist after development in auditory hair cells, they are retained in adult vestibular hair cells and contribute to bundle movement and mechanotransduction. Kinocilia in conditional *Camsap3* knockout mice lack the central microtubule pair that characterizes the “9+2” configuration in primary cilia. The kinocilia are also shorter, which suggests that CAMSAP3 may be required for the development and maintenance of these long cytoskeletal structures on vestibular hair cells. Another paper by Liu and Zhao studied the glutaredoxin domain-containing cysteine-rich family of proteins implicated in DFNB25/101. Their previous work showed that GRXCR2 is required for localizing taperin at the base of the stereocilia. Although mice lacking GRXCR2 are deaf and their hair bundles disorganized, the phenotype was rescued by reducing taperin expression. The current report indicates that GRXCR1 is also important for stereocilia morphogenesis, but it is distributed all along the stereocilia. In contrast to GRXCR2, it does not bind taperin. Hence, the change in hair bundle morphology and the hearing loss in *Grxcr1*-deficient mice are not rescued by decreasing taperin expression. Qi et al. investigated complement C1q Like 1 (C1QL1), which enables signaling receptor binding activity and is involved in formation and maintenance of synapse structure (Kakegawa et al., 2015; Sigoillot et al., 2015). This gene is of interest given that *C1ql1* knockout mice exhibit progressive hearing loss and OHC death. Although the number of auditory nerve fibers innervating inner and outer hair cells (IHCs, OHCs) decreases, the spiral ganglion neurons and the thickness of the myelin sheath are wildtype-like. In addition, the morphology, number, and function of IHCs are not affected in *C1ql1* knockout mice consistent with preferential expression of this gene in OHCs (Li et al., 2018). Based on these results, it is suggested that *C1ql1* may play a role in age-related hearing loss and/or the greater susceptibility of OHCs to various insults.

Hair cell regeneration was also addressed in this collection, with the aim of eventually recreating new, functional hair cells. In one report, a new zebrafish model was developed where the human diphtheria toxin receptor (hDTR) is expressed only in hair cells (Jimenez et al.). This approach allows for *in vivo* hair cell ablation in embryonic or adult zebrafish. In contrast to aminoglycoside administration or noise exposure, the injection of DT produces a synchronous destruction of all hair cells with limited effects on neighboring inner ear cells. Characterization of dose responses and the time course of regenerative responses are also documented. In the mammalian cochlea, Heuermann et al. report that regenerated hair cells in the lateral compartment of neonatal mice are innervated, but they simultaneously express markers for both outer and inner hair cells, i.e., oncomodulin *(Ocm)* and vesicular glutamate transporter 3 (*VGlut3*), respectively. It is, therefore, possible that cochlear hair cells, regenerated postnatally, may suffer from a delay or inability to differentiate into OHCs due to the alteration of signaling gradients that normally instruct such fates during development. Finally, Rudolf et al. provide evidence suggesting that the reduced proliferative capacity in mammalian utricular supporting cells may relate to the fact that circumferential F-actin bands are thicker than in their avian counterparts where hair cell regeneration is well documented. Differences in the stiffness and contractility of cytoskeletal elements that reinforce junctions between neighboring supporting cells may impact epithelial repair by reducing mechanical forces that evoke proliferation. In contrast to mice, chicken utricles remain relatively compliant, facilitating the dynamic mechanical signals produced during hair cell loss, and promoting their regeneration.

The possibility of developing effective interventions to mitigate hearing loss is supported by work from Chen et al.. Transcriptome-wide gene networks are shown to reflect shared molecular circuits and regulators in mouse models of sensorineural hearing loss due to aging, noise exposure and ototoxic drugs. The potential to use a single therapeutic approach to minimize the hearing loss associated with various etiologies would be most welcome. Mechanisms underlying aminoglycoside ototoxicity are also reviewed (Fu et al.). In addition to mitochondrial DNA mutations that increase susceptibility, over expression of NMDA receptors and the formation of free radicals also play a role. Administration of aminoglycosides increases the entry of calcium ions via NMDA receptors, ultimately resulting in neuronal cell death. Hence, glutamate-like excitotoxity can be induced by aminoglycosides. A better understanding of the underlying mechanisms will guide the development of interventions to reduce hearing loss due to ototoxic drugs, facilitating the transition from animal research to clinical practice. Lastly, given the current interest in synaptopathy, the development of more sophisticated diagnostic tools may help to categorize individuals with this malady and to better track their response to various therapeutics. Hence, the report by Bao et al. may provide a reliable metric for detecting synapse loss. Rather than measuring decreases in the Wave I ABR amplitude, they report greater reliability when quantifying curvature of the Wave I peak produced by clicks in mice pre- and post-noise exposure. If this approach can be validated in humans, it may provide an additional metric for clinical diagnoses.

Future studies are required to further elucidate the processes that guide hair cell differentiation, regeneration, and repair. The results of these endeavors will foster the development of new interventions that mitigate or prevent hearing loss and deafness.

## Author contributions

All authors listed have made a substantial, direct, and intellectual contribution to the work and approved it for publication.

## Conflict of interest

The authors declare that the research was conducted in the absence of any commercial or financial relationships that could be construed as a potential conflict of interest.

## Publisher's note

All claims expressed in this article are solely those of the authors and do not necessarily represent those of their affiliated organizations, or those of the publisher, the editors and the reviewers. Any product that may be evaluated in this article, or claim that may be made by its manufacturer, is not guaranteed or endorsed by the publisher.
